# Knockdown of HE4 suppresses tumor growth and invasiveness in lung adenocarcinoma through regulation of EGFR signaling

**DOI:** 10.32604/or.2024.045025

**Published:** 2024-05-23

**Authors:** YUE ZHANG, WENYU YANG, XIAOWANG HAN, YUE QIAO, HAITAO WANG, TING CHEN, TIANYING LI, WEN-BIN OU

**Affiliations:** 1 Department of Biopharmaceutics, College of Life Sciences and Medicine, Zhejiang Provincial Key Laboratory of Silkworm Bioreactor and Biomedicine, Zhejiang Sci-Tech University, Hangzhou, 310018, China; 2 Department of Thoracic Surgery, Zhejiang Provincial People’s Hospital, Hangzhou, 310014, China

**Keywords:** Lung adenocarcinoma, Human epididymis protein 4, Epidermal growth factor receptor, Biomarker, Targeted therapies

## Abstract

It has been shown that the high expression of human epididymis protein 4 (HE4) in most lung cancers is related to the poor prognosis of patients, but the mechanism of pathological transformation of HE4 in lung cancer is still unclear. The current study is expected to clarify the function and mechanism of HE4 in the occurrence and metastasis of lung adenocarcinoma (LUAD). Immunoblotting evaluated HE4 expression in lung cancer cell lines and biopsies, and through analysis of The Cancer Genome Atlas (TCGA) dataset. Frequent HE4 overexpression was demonstrated in LUAD, but not in lung squamous cell carcinoma (LUSC), indicating that HE4 can serve as a biomarker to distinguish between LUAD and LUSC. HE4 knockdown significantly inhibited cell growth, colony formation, wound healing, and invasion, and blocked the G_1_-phase of the cell cycle in LUAD cell lines through inactivation of the EGFR signaling downstream including PI3K/AKT/mTOR and RAF/MAPK pathways. The first-line EGFR inhibitor gefitinib and *HE4 shRNA* had no synergistic inhibitory effect on the growth of lung adenocarcinoma cells, while the third-line EGFR inhibitor osimertinib showed additive anti-proliferative effects. Moreover, we provided evidence that HE4 regulated EGFR expression by transcription regulation and protein interaction in LUAD. Our findings suggest that HE4 positively modulates the EGFR signaling pathway to promote growth and invasiveness in LUAD and highlight that targeting HE4 could be a novel strategy for LUAD treatment.

## Introduction

Lung cancer is the most common cause of cancer-related death worldwide. 2.1 million people are diagnosed with lung cancer each year, and 1.8 million people die from the diseases [1]. The 5-year survival rate depends on stage and regional differences, ranging from 4% to 17%. Lung cancers are classified as non-small cell lung cancer (NSCLC, ~85%) and small cell lung cancer (SCLC, ~15%) [2]. NSCLC is believed to originate from lung epithelial cells and encompasses a variety of histological subtypes, including lung adenocarcinoma (LUAD), lung squamous cell carcinoma (LUSC), and large cell carcinoma (LCC) [3]. The most common genetic variants in NSCLC mainly include epidermal growth factor receptor (*EGFR*) mutations, echinoderm microtubule-associated protein-like 4-anaplastic lymphoma kinase (*EML4-ALK*) gene fusion, tyrosine kinase hepatocyte growth factor receptor (*HGFR*, also known as *MET*) amplification, Kirsten rat sarcoma (*KRAS*) mutations, *ROS1* proto-oncogene rearrangement, rearranged during transfection (*RET)* gene fusion, and inactivation of tumor suppressor P53 and liver kinase B1 (LKB1) [4–8].

EGFR has become a crucial therapeutic target for the treatment of NSCLC [5]. Activation of mutated EGFR and downstream intermediates—including RAF/MAPK, and PI3K/AKT/mTOR—eventually leads to increased proliferation and migration in NSCLC [5,9]. The activation of EGFR may be dysregulated by a variety of carcinogenic mechanisms, including *EGFR* mutations, amplification, and protein overexpression [10]. Various studies have shown that the deletion of EGFR exon 19 (del) and mutation of L858R on exon 21 in most NSCLC cells are sensitive to EGFR inhibitors (gefitinib, erlotinib, afatinib, and dacomitinib). In the end, 50%–65% of tumor patients harboring these EGFR mutations acquire the above-mentioned inhibitor resistance due to *EGFR* T790M. This mutation prompted the development and application of third-generation EGFR inhibitors, osimertinib and rositinib (rociletinib), to the clinical treatment of patients with drug-resistant lung cancer [11–14]. In addition, when compared with patients with L858R mutations, T790M mutations occur more frequently in patients with deleted mutations in *EGFR* exon 19 [12].

Human epididymis protein 4 (HE4, other name WFDC2) was originally found in epithelial cells in the epididymis, and it is speculated that the function of HE4 is to act as a protease inhibitor during sperm maturation [15]. HE4 is a glycosylation protein [16], mainly located in the cell membrane and cytoplasm, and can also be detected in the perinuclear region [17]. Various studies have shown that HE4 functions as one biomarker in different cancers, including ovarian cancer [18], lung cancer [19,20], endometrial cancer [21], and breast cancer [22]. In addition, increasing evidence suggests that HE4 regulates cell proliferation and metastasis in ovarian cancers. HE4 silencing inhibits proliferation and migration in ovarian cancer cells through the regulation of PI3K/AKT, RAF/MAPK, and JAK/STAT3 signaling pathways [23,24]. Further data demonstrated that the interaction of HE4 and YWHAE, ZNF703, or ANXA2 mediated ovarian cancer growth and metastasis [25–27].

A variety of data have indicated that HE4 could serve as a diagnostic biomarker in lung cancer [28,29], but the function and mechanisms of HE4 in LUAD are poorly understood. In the current study, as compared to adjacent normal tissues, overexpression of HE4 was found in LUAD, but not in LUSC, which has been reported in a previous study [30]. *HE4 shRNA* knockdown resulted in anti-proliferative effects in LUAD, which was associated with the interaction of HE4 and EGFR, downregulation of EGFR, and inactivation of downstream PI3K/AKT/mTOR and RAF/MAPK signaling pathways. These novel findings indicate that HE4 might be a biologically rational target for LUAD treatment, especially in first/second-generation EGFR inhibitor-resistant lung cancer.

## Materials and Methods

### Experimental methods

Lentivirus preparation and infection, immunoblotting, co-immunoprecipitation, colony formation, cell cycle, apoptosis, wound healing, and transwell matrigel assays have been previously described [31,32]. All the assays were carried out from triplicate experiments.

### Antibodies and reagents

All the information on primary antibodies is shown in the Suppl. Table S1. Primary antibodies to EGFR (western, sc-03), EGFR (co-IP, sc-120), and HE4 (co-IP, sc-293473), and protein G beads were obtained from Santa Cruz Biotechnology (Santa Cruz, CA, USA). Anti-GAPDH and anti-actin antibodies were obtained from Absin Bioscience Inc. (Shanghai, China), and polyclonal rabbit anti-HE4 antibody was homemade. Phosphorylation antibodies to EGFR (Y1068), AKT (S473), and MAPK (T202/Y204) were obtained from Cell Signaling Technology (Beverly, MA, USA). Trizol was purchased from Invitrogen Life Technologies (Carlsbad, CA, USA). Crystal violet, Puromycin, and polybrene were from Solarbio Life Sciences (Shanghai, China). The PE Annexin V Apoptosis Detection Kit I was from BD Pharmingen (San Jose, CA, USA). PolyJet was purchased from Signagen (Jinan, China). MTT, bovine serum albumin (BSA), and Lentiviral *HE4 shRNA* constructs were from Sigma-Aldrich (St, Louis, MO, USA). DAPI was from Beyotime Biotechnology (Shanghai, China). Gefitinib and osimertinib were obtained from Selleck Chemicals LLC (Shanghai, China). All inhibitors were reconstituted in DMSO (Sigma-Aldrich, Louis, MO, USA).

### Cell lines, cell culture, and tumor specimens

Three representative LUAD cell lines were used for HE4 functional studies, each of which has a different EGFR mutation status. PC-9 harbors *EGFR* exon 19 deletion (E746-A750), which is sensitive to the first/second generation inhibitors of EGFR. H1975 harbors *EGFR* L858R mutation in exon 21 and T790M mutation in exon 20, which is strongly resistant to the first/second generation inhibitors of EGFR. A549 is not sensitive to the first/second/third generation EGFR inhibitors because of its wild-type EGFR profile. PC-9, H1975, and A549 cells were cultured in RPMI-1640 (Gibco) supplemented with 10% fetal bovine serum (FBS), 1% penicillin/streptomycin, and 1% L-glutamine in 5% CO_2_ at 37°C. Cell lines were regularly screened using the Mycoplasma Stain Assay Kit (Beyotime Biotechnology, Shanghai, China).

Normal lung cell Beas-2B and LUAD cell lines (A549, H1975, PC-9, and H661) are kind gifts from Dr. Li Chai at Brigham and Women’s Hospital, Harvard Medical School. LUSC cell lines (SK-MES-1 and NCI-1703) and LUAD cell lines (SPC-A1 and LTEP-A2) are kind gifts from Dr. Hongbin Ji at Shanghai Institute of Biochemistry and Cell Biology (Shanghai, China), Dr. Zhaohui Gong at Ningbo University (Ningbo, China), respectively.

All lung cancer frozen tumor samples and adjacent non-neoplastic specimens were discarded tissues. The studies were conducted in accordance with recognized ethical guidelines (U.S. Common Rule), were approved by Zhejiang Provincial People’s Hospital, and Zhejiang Sci-Tech University Institutional Review Boards (#20220304). The patients provided their written informed consent to participate in this study.

### Lentiviral HE4 shRNA constructs and virus infection

Lentiviruses were prepared by co-transfection of pLKO/*HE4 shRNA* constructs, and Δ8.9 and VSVG into 293T cells using PolyJet. Lentiviruses were harvested at 24, 36, 48, and 60 h post transfection, and frozen at −80°C in aliquots. The seeded cells were infected by lentiviral shRNAs in the presence of 10 ng/μL polybrene. Cells were selected with 0.75 μg/mL puromycin to generate lines with stable *HE4 shRNA* expression.

### Protein lysate preparations and immunoblotting

The whole cell lysates were prepared using lysis buffer as our previous published report [32]. Frozen tumor tissues and adjacent noncancerous tissues were homogenized in cold lysis buffer and the cell lysates were then rocked overnight at 4°C. Electrophoresis and immunoblotting were carried out as described previously [33]. The protein signals were detected by ECL (Millipore Corporation, MA, USA), and captured using a Tanon5500 chemiluminescence imaging system (Tanon Bio-Sciences Corporation, Shanghai, China).

### Co-immunoprecipitation

In brief, 2 μg of anti-HE4 or anti-EGFR antibody was incubated with pre-cleared lysates (1 mg) using 20 μL of sepharose-protein G beads for 2 h at 4°C. Then 25 µL of protein G beads were incubated with primary antibody and lysates overnight at 4°C. The interactions of HE4 and EGFR were evaluated by co-immunoprecipitation and immunoblotting (HE4 and EGFR) after the protein G beads were washed four times (three times with 750 µL of IP buffer and once with 750 µL 10 mM Tris-Cl buffer (pH 7.4)).

### Cell viability analysis

The plated LUAD cell lines (H1975, A549, and PC-9) (3000 cells/well) in a 96-well plate (Greiner, Frickenhausen, Germany) were infected with lentiviral *HE4 shRNAs*, then treated 24 h later with EGFR inhibitor gefitinib or osimertinib. Cell proliferations were analyzed at 3, 6, or 9 days using the MTT approach, and quantified using a Microplate Reader (TECAN, Austria) after treatment with lentiviral *HE4 shRNAs* and/or inhibitors in three LUAD cell lines or HE4-silenced H1975 and A549 with stable shRNA expression.

### Colony-formation assay

The stably silenced HE4 H1975 and A549 cells were plated (3000 cells/well) in six-well plates and cultured until there were 30–50 individual cells of the clone. PC-9 cells were infected with lentiviral *HE4 shRNAs* for 5 days after these cells (3000 cells/well) were plated and cultured for 2 days. The fixed (methanol) and stained (0.1% crystal violet) colonies were dissolved using 900 µL of 33% acetic acid solution and A_570_ was measured after the colonies were photographed.

### Cell cycle analysis

LUAD cells in six-well plates were trypsinized and washed with PBS buffer at 6 days post-infection with lentiviral *HE4 shRNAs* in PC-9 cells or in H1975 cells with stably expressed *HE4 shRNAs*. The fixed (pre-cooled 70% ethanol) and stained (PI solution) cells were measured in Accuri C6 (BD Biosciences, NJ, USA). Data was analyzed using Flow Jo and CFlow Plus.

### Apoptosis assay

The stained PC-9 cells with PE Annexin V and 7-AAD at 6 days post-infection with lentiviral *HE4 shRNAs* were measured in Accuri C6 (BD Biosciences, NJ, USA) within 2 h and the data were analyzed by CellQuest software (BD Biosciences).

### Wound healing assays

A wound was created using the tip of a P-100 pipetman in near-confluent H1975 and A549 with stable *HE4 shRNA* expression, and at 4 days post-infection with lentiviral *HE4 shRNAs* in near-confluent PC-9. Would healing status was photographed at hours 0, 24, or 72 using a Leica DMI 3000B inverted microscope (Leica Microsystems, Germany).

### Transwell matrigel assays

H1975 and A549 cells (7 × 10^4^) with stable *HE4 shRNA* expression and PC-9 cells (7 × 10^4^) infected with lentiviral *HE4 shRNAs* for 48 h were seeded on the upper chamber. After 48 h, the fixed (100% methanol) and stained (0.1% crystal violet) invasive cells were photographed using an inverted microscope (Leica DMI 3000 B Microsystems, Germany), and quantified the OD value at 570 nm after treatment with 33% acetic acid.

### Immunofluorescence staining

The immobilized (4% formaldehyde) and permeabilized (100% methanol) PC-9 cells (1 × 10^6^) were incubated with mouse monoclonal HE4 (sc-293473) and rabbit polyclonal EGFR (sc-120) in 5% BSA overnight at 4°C after blocking using 5% BSA. And then these cells were incubated with Alexa fluor 488 or 595 secondary antibodies (Invitrogen Molecular Probes, Carlsbad, CA, USA) after washing using PBS. The cells were photographed using a laser confocal microscope (TCS SPE ll, Leica, Germany) after washing and staining (DAPI).

### Quantitative real-time polymerase chain reaction

Quantitative real-time polymerase chain reaction (qRT-PCR) assays were performed on Applied Biosystems 7500 Real-Time PCR (Carlsbad, CA, USA) using SYBR Premix Ex Taq™ (Takara) after cDNA was synthesized using PrimeScript 1st Strand cDNA Synthesis Kit (Takara, Beijing, China). The levels of *HE4* mRNA were calculated by the 2^−ΔΔCT^ method and *GAPDH* was used as an internal control. The qRT-PCR assays were carried out using the following primers: *HE4* primers were purchased from Gene Copoeia (#HQP000481, Guangzhou, Guangdong, China). *GAPDH* primer sequences have been reported in our previous study [32]. *EGFR* sense: AGCAGAGACCCACACTACCA; *EGFR* anti-sense: GTAGTCAGGGTTGTCCAGGC.

### Statistical analysis

Statistically significant differences between control and treatment were analyzed using student’s *t*-tests and were defined as *p < 0.05, **p < 0.01, ***p < 0.001, and ****p < 0.0001.

## Results

### HE4 upregulation in LUAD, but downregulation in LUSC

**Figure 1:**
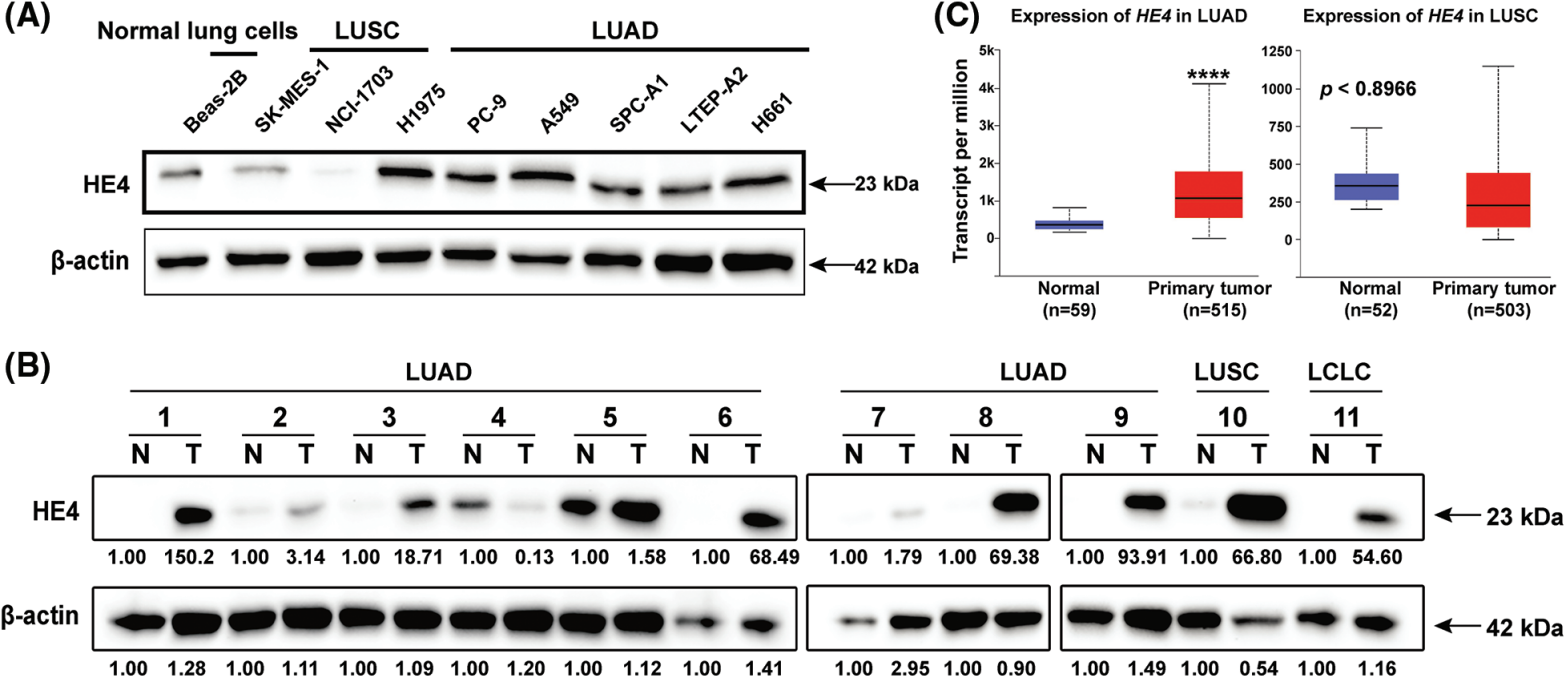
HE4 overexpression is present in LUAD, but not in LUSC. (A) Immunoblotting demonstrates strong HE4 expression in LUAD cell lines compared with the Beas-2B. (B) Immunoblotting demonstrates overexpression of HE4 in ten of eleven lung cancer biopsies, as compared to adjacent non-neoplastic tissue samples. (C) TCGA gene expression data analysis on 503 LUSC and 515 LUAD shows that *HE4* expression is higher in LUAD patient samples, and lower in LUSC patient samples compared to adjacent normal tissues. ****p < 0.0001.

HE4 expression was first evaluated by immunoblotting in Beas-2B normal lung cell line and eight NSCLC lines (SK-MES-1, NCI-1703, H1975, PC-9, A549, SPC-A1, LTEP-A2, and H661) (Fig. 1A). Immunoblotting demonstrated that HE4 is upregulated in LUAD cell lines, but downregulated in LUSC lines, compared to Beas-2B. Overexpression of HE4 was further demonstrated in ten of eleven lung cancer biopsy samples (nine cases of LUAD, one case of LUSC, and one case of LCLC), as compared with adjacent normal tissues (Fig. 1B). In the Cancer Genome Atlas (TCGA) database (Ualcan.path.uab.edu/analysis), we analyzed the expression of *HE4* in lung cancer: Compared with adjacent normal tissues, HE4 was significantly overexpressed in LUAD patients (n = 515), whereas there is opposite expression in LUSC (n = 503) (Fig. 1C).

### HE4 knockdown inhibits proliferation, and colony formation, and induces apoptosis in LUAD cell lines

**Figure 2:**
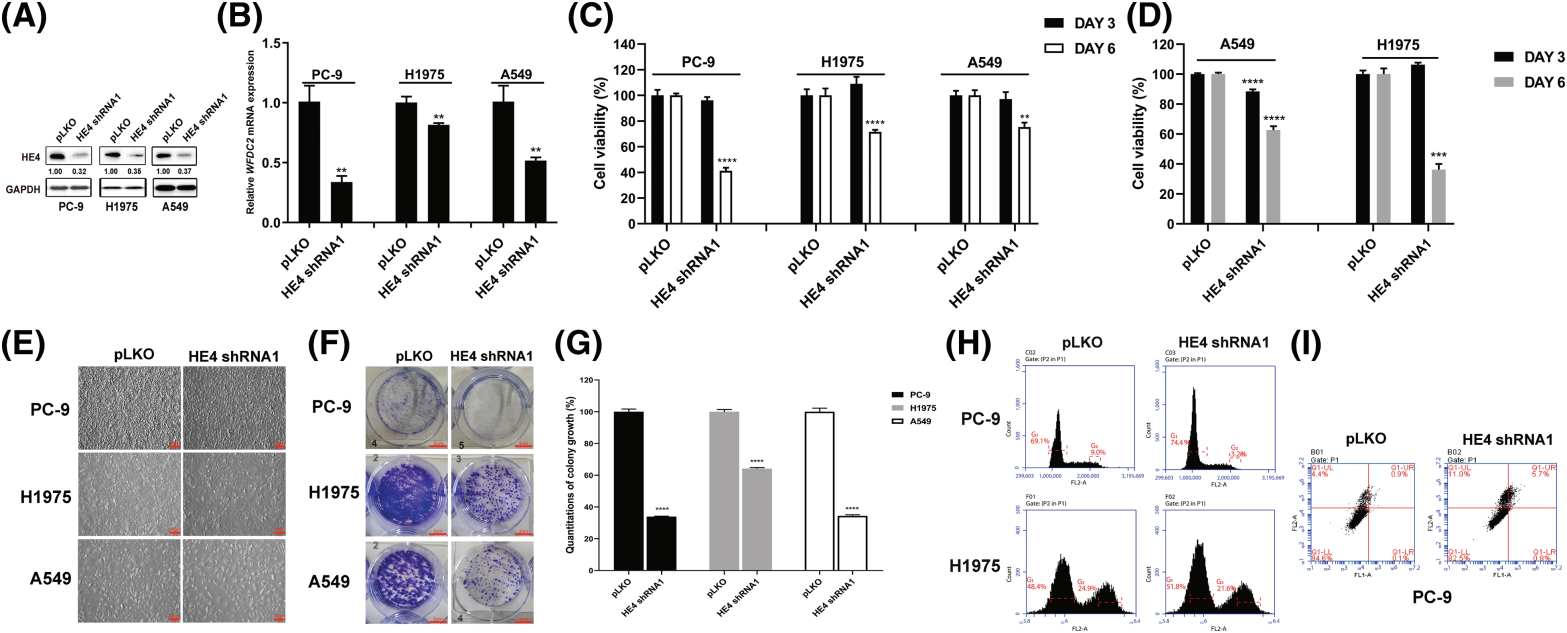
HE4 knockdown inhibits proliferation, and colony formation, and induces apoptosis in LUAD cell lines. (A) Immunoblotting shows HE4 silencing. (B) Quantitative RT-PCR evaluations of HE4 (*WFDC2*) transcripts in PC-9 at day 6 post-infection with *HE4 shRNA1*, in H1975, and A549 cells with stable lentiviral *HE4 shRNA1* construct. (C) Cell viability was evaluated by an MTT assay after infection with lentiviral *HE4 shRNA1*. (D) Cell viability was evaluated by an MTT assay in H1975 and A549 cells with stable *HE4 shRNA1* expression. (E) Cell culture appearance was evaluated after treatment with *HE4 shRNA1*. Scale bars: 100 µm. (F) Colony growth assays were performed after treatment with *HE4 shRNA1*. Scale bars: 100 μm. (G) Quantitation (A_570_) of colony growth after treatment with *HE4 shRNA1*. (H) Cell cycle assay was carried out in PC-9 at day 6 post-infection with *HE4 shRNA1*, and in H1975 cells with stable *HE4 shRNA1* expression. (I) Apoptosis assays were performed at day 6 post-infection of *HE4 shRNA1*. **p < 0.01; ***p < 0.001; ****p < 0.0001.

HE4 expression was knocked down by lentiviral shRNAs in LUAD cell lines (PC-9, H1975, and A549). The immunoblotting evaluation showed that *HE4 shRNA1* knockdown led to about a 70% reduction of target protein expression (Fig. 2A). The *HE4* (*WFDC2*) silencing at the transcriptional level was verified by qRT-PCR (Figs. 2B and S1A). The results showed that lentiviral *HE4 shRNA* dramatically downregulated the expression of HE4 in these LUAD cell lines, as compared with the control empty vector pLKO.

Three LUAD cell lines were transiently infected with *HE4 shRNAs*, and cell viability was measured at 3- and 6-days post-infection by MTT assays (Fig. 2C and Suppl. Fig. S1B). Compared with the control group pLKO, HE4 knockdown markedly inhibited cell viability in PC-9, H1975, and A549 cell lines. *HE4 shRNA1* knockdown reduced viability at day 6 by 60% in PC-9 and by 30% in H1975 and A549 (Fig. 2C). *HE4 shRNA2* knockdown reduced viability at day 6 by 80% in PC-9, by 45% in H1975, and by 50% in A549, respectively (Suppl. Fig. S1B). Furthermore, the stable *HE4 shRNA1* knockdown at day 6 resulted in 40% and 70% viability reduction in A549 and H1975, and *HE4 shRNA2* knockdown at day 6 inhibited cell viability by 30% and 60% in A549 and H1975, as compared to the pLKO control (Fig. 2D and Suppl. Fig. S1C).

Cell growth of H1975 and A549 cells with stably expressing *HE4 shRNAs*, and PC-9 at 6 days post-infection with *HE4 shRNAs*, was dramatically reduced, when evaluated by six-well monolayer culture (Fig. 2E and Suppl. Fig. S1D). The cell growth status of the *HE4 shRNAs* was markedly different from that of the pLKO control. The adhesion of cells has deteriorated; a large number of cells fell off, and cell morphology became rounded in PC-9. The adherence ability of H1975 cells was weakened, as evidenced by the shortened trypsinization time of cells. Compared to H1975 and PC-9, A549 cells were larger, grew more slowly, and became less adherent.

Colonies of LUAD cells (PC-9, H1975, and A549) infected with *HE4 shRNAs* were fewer and smaller compared to the pLKO control (Fig. 2F and Suppl. Fig. S2A), colony formation reduced by approximately 70%–80% in PC-9 cells, 30%–40% in H1975 cells, and 55%–65% in A549 (Fig. 2G and Suppl. Fig. S2B).

The stable *HE4 shRNA* knockdown in PC-9 led to an increase of G_1_ population, from 69.1% in the pLKO-infected control to 74.4% in *HE4 shRNA1* or 92.5% in *HE4 shRNA2*-treated cells, and a decrease in the G_2_ phase population from 9.0% with the pLKO control to 3.2% with *HE4 shRNA1* and 1.4% with *HE4 shRNA2*. *HE4 shRNA* knockdown in H1975 also increased the G_1_ peaks, from 48.4% in the pLKO-infected cells to 51.8% in *HE4 shRNA1* or 61.4% in *HE4 shRNA2*-infected cells and reduced the G_2_ phase population from 24.9% with the pLKO control to 21.6% with *HE4 shRNA1* and 13.4% with *HE4 shRNA2* (Fig. 2H and Suppl. Fig. S3A).

In apoptosis assays, *HE4 shRNA* knockdown resulted in an increase in apoptotic cells at 5 days post-infection in PC-9, from 5.4% in the pLKO-treated cells to 17.5% in *HE4 shRNA1* or 32.4% in *HE4 shRNA2*-treated cells (Fig. 2I and Suppl. Fig. S3B), however, the statistical analysis does not show a significant difference between control and HE4 knockdown groups.

### HE4 knockdown blocks migration and invasiveness in LUAD cells

**Figure 3:**
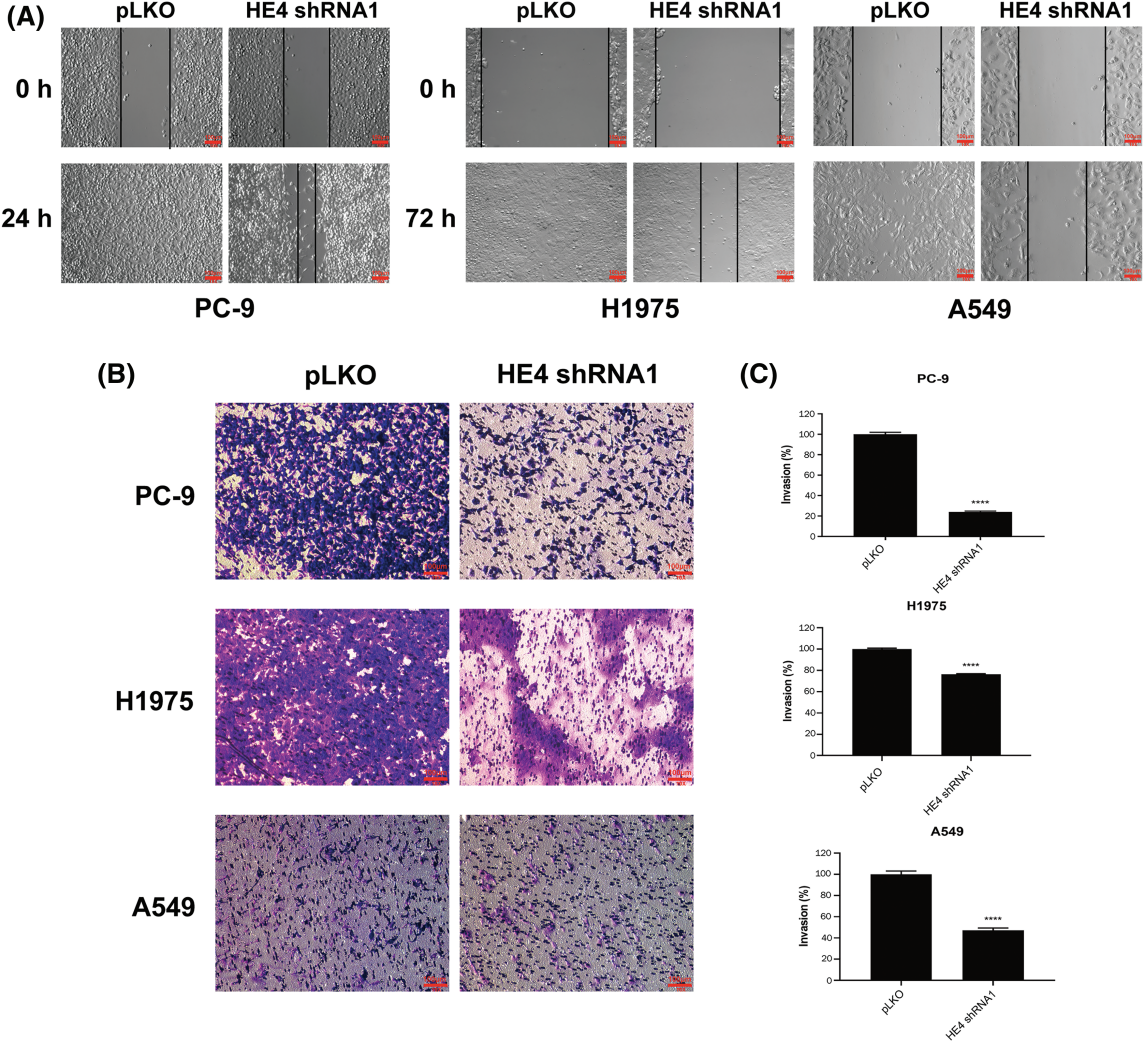
HE4 knockdown suppresses migration and invasiveness in LUAD cells. Wounding assays (A) and transwell migration assays (B) were performed in PC-9, H1975, and A549 after HE4 shRNA1 knockdown. Scale bars: 100 μm. (C) Quantitation (A_570_) of LUAD cell invasiveness in PC-9 at day 6 post-infection of *HE4 shRNA1*, in H1975 and A549 cells with stable *HE4 shRNA1* expression. ****p < 0.0001.

Assays were performed in H1975, A549, and PC-9 to evaluate the anti-migration and -invasive effects of HE4 knockdown. Wound-healing assays demonstrated that *HE4 shRNA* knockdown impaired wound closure at 72 h in H1975 and A549, and at 24 h in PC-9, whereas full wound closure was observed in pLKO control cells (Fig. 3A and Suppl. Fig. S4A). Matrigel assays showed similar results, with about 30% and 60% inhibition of invasiveness in H1975 and A549 with stable *HE4 shRNA* expression, respectively, and about 85% inhibition after transient knockdown of HE4 in PC-9 cells, as compared with the pLKO control (Figs. 3B, 3C and Suppl. Figs. S4B, S4C). Both cell wound healing and transwell assays showed that HE4 overexpression promoted migration and invasion in lung cancer cells.

### Anti-proliferative and pro-apoptotic effects of HE4 knockdown in LUAD cells are associated with inhibition of EGFR signaling, down-regulation of EGFR, and interaction of EGFR with HE4

**Figure 4:**
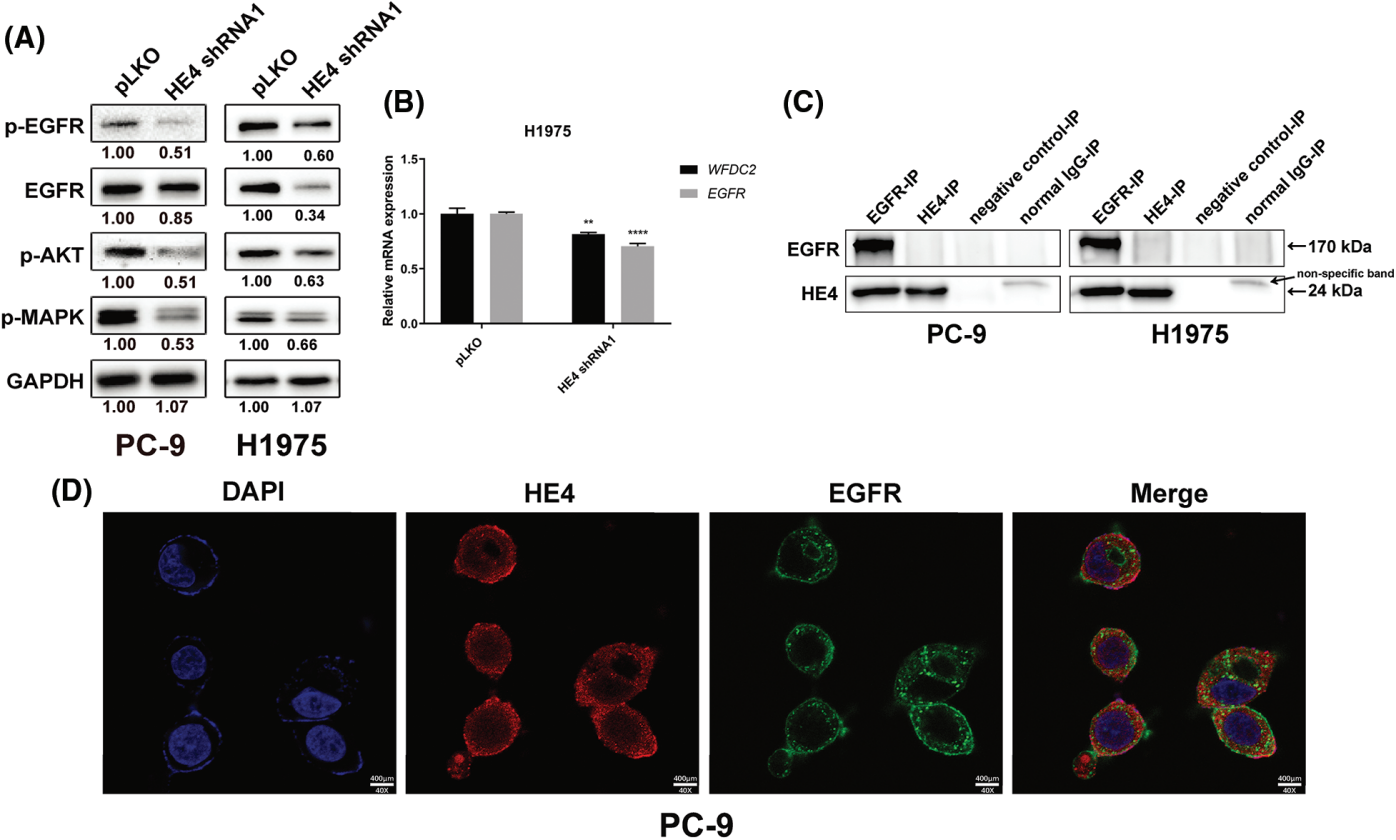
HE4 knockdown inhibits EGFR signaling, down-regulation of EGFR, and interaction of EGFR with HE4 in LUAD cells. (A) Immunoblotting analyzed expression of p-EGFR, EGFR, p-AKT, and p-MAPK in PC-9 at day 6 post-infection with *HE4 shRNA1*, and in H1975 cells with stable *HE4 shRNA1* expression. (B) QRT-PCR was carried out using *HE4* (*WFDC2*) and *EGFR* transcripts from H1975 cells with stable *HE4 shRNA1* expression. **p < 0.01; ****p < 0.0001. (C) EGFR and HE4 IPs followed by EGFR and HE4 stains in PC-9 and H1975 show interaction between HE4 and EGFR. (D) Cell location of HE4 (red color) and EGFR (green color) in PC-9 was evaluated by immunofluorescence staining. The orange color indicates the image merge of EGFR and HE4 stains. Scale bars: 400 μm.

EGFR signaling was evaluated by immunoblots in PC-9 at 6 days post-transduction of *HE4 shRNAs* and H1975 with stably silenced HE4 expression (Fig. 4A). HE4 knockdown inhibited EGFR expression and activation, and dephosphorylated AKT and MAPK in PC-9 and H1975 cell lines (Fig. 4A). The quantitation of EGFR signaling expression is also shown in Fig. 4A. Furthermore, HE4 knockdown inhibited *WFDC2* and *EGFR* transcripts in the H1975 cell line with stable *HE4 shRNA* expression (Fig. 4B and Suppl. Fig. S5).

To evaluate the interaction of HE4 and EGFR, we performed EGFR and HE4 immunoprecipitations (co-IP), followed by EGFR and HE4 immunoblotting in PC-9 and H1975 (Fig. 4C). The EGFR IPs revealed an EGFR 170 kDa and a HE4 24 kDa band in PC-9 and H1975, which was confirmed by EGFR and HE4 stains in EGFR co-IPs (Fig. 4C). The results showed that HE4 may interact with EGFR. To further confirm the interaction of HE4 with EGFR, immunofluorescence staining experiments were performed in PC-9 cells. Results showed that HE4 (red color) expression was predominantly in the cell membrane, cytoplasm, and nucleus, whereas EGFR (green color) expression was in the cell membrane and cytoplasm, with co-localization of HE4 and EGFR in the cell membrane and cytoplasm (Fig. 4D).

### Additive anti-proliferative effects of combination treatment with osimertinib and HE4 shRNAs in EGFR mutated lung cancer cell lines

**Figure 5:**
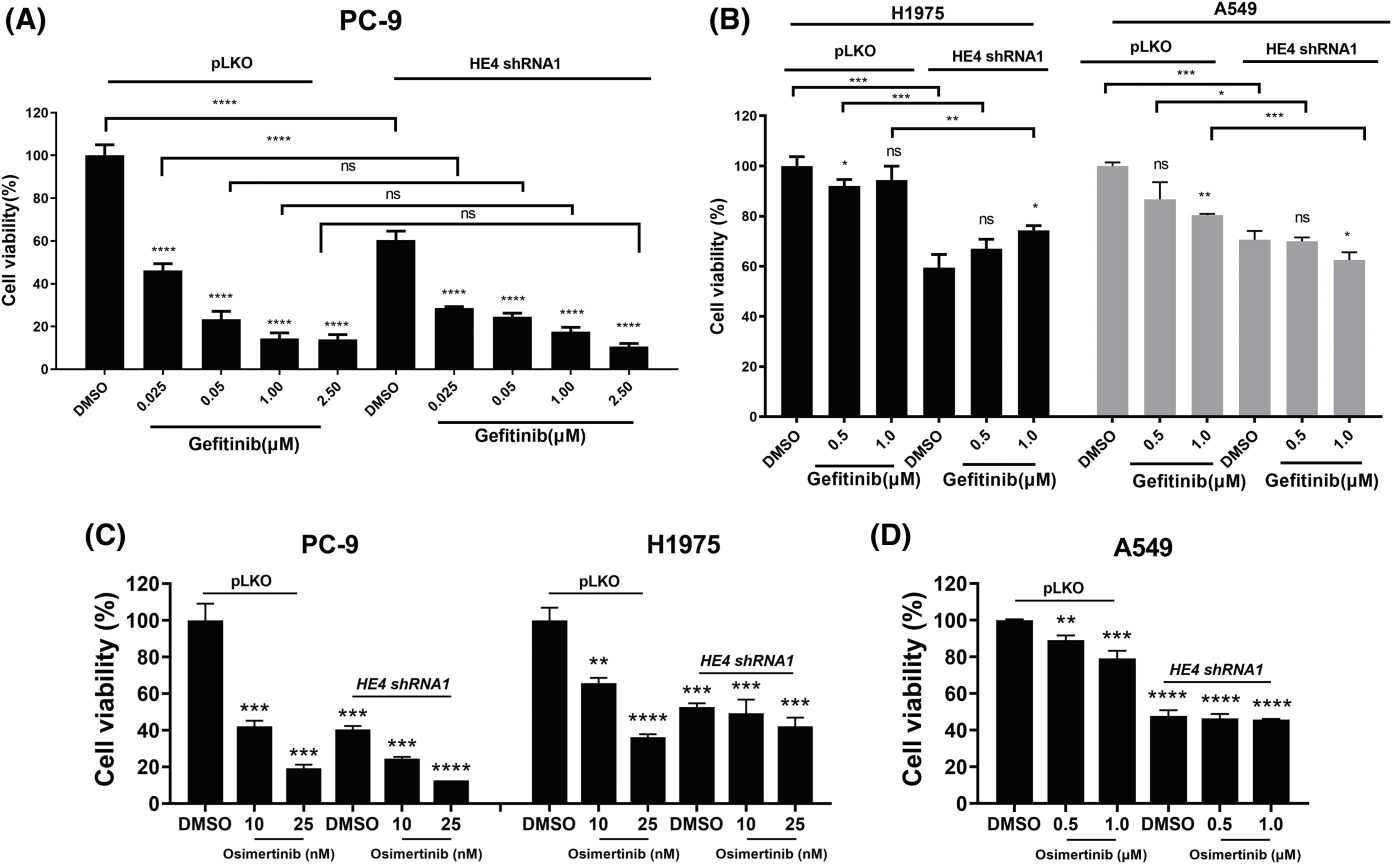
Additive anti-proliferative effects of combination treatment with gefitinib/osimertinib and *HE4 shRNA1* in LUAD cell lines. Cell proliferation was measured by an MTT assay in PC-9, H1975, and A549 after combination treatment with *HE4 shRNA1* and gefitinib (A and B) /osimertinib (C and D) together or alone for 6 days. ns: *p* ≥ 0.05; *p < 0.05; **p < 0.01; ***p < 0.001; ****p < 0.0001.

Additive anti-proliferative effects were observed after combined inhibition of EGFR (gefitinib and osimertinib) and HE4 (shRNAs) in PC-9, H1975, and A549 (Fig. 5 and Suppl. Fig. S6). Additive mild anti-proliferative effects were seen in PC-9 (low doses of gefitinib) and A549 (high doses of gefitinib) cell lines after the combination of *HE4 shRNAs* and gefitinib treatment, but not in the H1975 cell line (Figs. 5A, 5B, Suppl. Figs. S6A and S6B). Additive anti-proliferative effects were seen in PC-9 and H1975 cell lines after the combination of *HE4 shRNAs* and osimertinib treatment, but not in the A549 cell line (Figs. 5C, 5D, Suppl. Figs. S6C and S6D). Combinational inhibiton of HE4 and EGFR by shRNA and osimertinib led to greater viability reduction in PC-9 and H1975, as compared with *HE4 shRNAs* or osimertinib treatment alone (Figs. 5C, 5D, Suppl. Figs. S6C and S6D).

## Discussion

The oncogenic receptor tyrosine kinases are key to lung cancer malignant transformation, as evidenced by the clinical successes of EGFR, MET, and EML4-ALK inhibition by small molecule therapeutics [7,8]. Although the clinical success of TKIs has been dramatic, specifically for the first/second/third EGFR inhibitors, most lung cancer patients finally encounter disease progression because of various TKI resistance mechanisms [4,5,9]. Such mechanisms include *EGFR/ALK* novel mutations, *MET/ERBB2* amplification, *NRG1* fusion, IGF1R activation, and Hippo pathway inhibition, and so on. To substantially improve lung cancer patient survival, the identification of novel biologically rational targets will be required.

High levels of HE4 expression have been found in NSCLC, and most studies focus on the biomarker roles of HE4 in lung cancer diagnosis [19,20,28], involving differences such as gender, race, and tumor progressive stage in NSCLC and SCLC. However, the mechanism of pathological transformation of HE4 in lung cancer is still unknown. Thus, in the current report, the roles of HE4 expression and biological function were investigated in NSCLC.

The immunoblotting evaluation demonstrated that HE4 was highly expressed in NSCLC cell lines, as compared to normal lung cells (Fig. 1A). Overexpression of HE4 was also found in 10 of 11 lung cancer biopsy samples, including nine LUAD, one LUSC, and one LCLC, as compared with adjacent normal tissues (Fig. 1B). TCGA dataset analysis also found that HE4 expression was stronger in LUAD, and weaker in LUSC than that of normal samples (Fig. 1C). An earlier study has reported that HE4 expression is negative in most of the LUSC cases (n = 41), just one case is positive, whereas HE4 is highly expressed in most of the LUAD cases [30]. Taken together, these data indicate that HE4 overexpression may contribute to tumorigenesis and that elevated levels of HE4 predict poor prognosis in LUAD. HE4 may play a tumor suppressor role in LUSC, but more data is needed to illustrate. This is the implication of HE4 function complexity in NSCLC. Therefore, the correlation between HE4 expression and important NSCLC clinicopathologic variables need be studied in larger cohorts in our following studies.

Recent studies have found that HE4 overexpression promotes proliferation and invasiveness in ovarian cancer [23,24]. We show that *in vitro* LUAD viability, colony formation, migration, and invasion are inhibited by *HE4 shRNA* knockdown (Figs. 2, 3 and Suppl. Figs. S1–S4) through down-regulation of EGFR, interaction of HE4 and EGFR, and inactivation of EGFR signaling (Fig. 4 and Suppl. Fig. S5), which are consistent with high HE4 expression correlated with poor prognosis in lung cancer [28]. However, *in vivo* model studies are needed to evaluate the anti-proliferative and anti-migration effects of targeting HE4.

The interaction/complex of HE4 and EGFR was found in PC-9 and H1975 by co-IPs and immunofluorescence stains (Figs. 4C and 4D), which may be associated with the high Lewis y antigen expression on HE4 and EGFR [16]. This is consistent with the previous finding that HE4 binds to the extracellular domain of EGFR in prostate cancer [34]. The lactose series of fucosyl glycans, Lewis glycans, have also been shown to be closely related to the malignant transformation of tumors [17]. In addition, the Lewis y antigen on the surface of HE4 regulates tumor invasion and metastasis. The Lewis y antigen expressed by lung cancer cells may change EGFR phosphorylation and participate in cell malignant behavior [35]. Studies have confirmed that Lewis y antigen not only exists in integrins α5 and β1 but also in EGFR [36]. The tyrosine phosphorylation of EGFR is enhanced because of overexpression of Lewis y antigen [16,36]. Although the current data indicates Lewis y antigen has a crucial function in EGFR and HE4 oncogenic roles, the interactions or complex of EGFR and HE4 related to the Lewis y antigen need to be studied in detail.

To investigate HE4 function and oncogenic mechanisms in NSCLC, three representative LUAD cell lines were used in the present study. Due to various EGFRi resistance mechanisms, we further evaluated whether there are additive anti-proliferative effects in these three LUAD cell lines after co-targeting HE4 and EGFR using *HE4 shRNAs* and gefitinib or osimertinib, specifically, in EGFRi resistant LUAD (Fig. 5 and Suppl. Fig. S6). HE4 knockdown markedly inhibited cell viability in three LUAD cell lines with different EGFR mutation status. These data imply that HE4 is one target candidate in LUAD with wild-type or mutated EGFR, especially, in the first/second generation EGFRi-resistant LUAD, which is associated with EGFR signaling inhibition. However, HE4 function roles need further evaluation against third-generation EGFRi resistance. Additive anti-proliferative effects were seen in PC-9 and H1975 cell lines after combination treatment with *HE4 shRNAs* and osimertinib, as compared with *HE4 shRNAs* or osimertinib treatment alone, but not in combination treatment with *HE4 shRNAs* and gefitinib. This data indicate that wild-type EGFR has no oncogenic role in lung cancer and targeting HE4 might be a novel strategy in EGFRi-sensitive and resistant LUAD therapies. The present results have demonstrated that HE4 oncogenic roles are related to EGFR signaling and regulation, and additive anti-proliferative effects further indicate HE4 function beyond EGFR regulation in LUAD.

## Supplementary Materials

Figure S1 **A**) QRT-PCR evaluations of *HE4* (*WFDC2*) in PC-9 at day 6 post-infection with *HE4 shRNA2*, in H1975, and A549 cells with stable lentiviral *HE4 shRNA2* construct. **B**) Cell viability was measured by an MTT assay in PC-9, H1975, and A549 at days 3 and 6 after infection with lentiviral *HE4 shRNA2*. **C**) Cell viability was evaluated by an MTT assay in H1975 and A549 cells with stable *HE4 shRNA2* expression at days 3 and 6. **D**) Cell culture appearance was evaluated in PC-9 at day 6 post-infection of *HE4 shRNA2*, in H1975, and A549 cells with stable lentiviral *HE4 shRNA2* construct. Scale bars: 100 µm. **p<0.01; ***p<0.001; ****p<0.0001.

Figure S2 **A**) Colony growth assays were carried out in PC-9 at day 6 post-infection with *HE4 shRNA2* and in H1975 and A549 cells with stable *HE4 shRNA2* expression. Scale bars: 100 μm. **B**) Quantitation (A570) of PC-9, H1975, and A549 cell colony growth after treatment with *HE4 shRNA2*. ***p<0.001; ****p<0.0001.

Figure S3 **A**) Cell cycle analysis was performed in PC-9 at day 6 post-infection with *HE4 shRNA2*, and in H1975 cells with stable *HE4 shRNA2* expression. **B**) Apoptosis assays were performed in PC-9 at day 6 post-infection of *HE4 shRNA2*.

Figure S4 HE4 knockdown suppresses migration and invasiveness in LADC cell lines. Wounding assays (**A**) and transwell migration assays (**B**) were performed after *HE4 shRNA2* knockdown. Scale bars: 100 μm. (**C**) Quantitation (A570) of LADC cell invasiveness in PC- 9 at day 6 post-infection of *HE4 shRNA2*, in H1975 and A549 cells with stable *HE4 shRNA2* expression. *p<0.05; **p<0.01; ****p<0.0001.

Figure S5 Quantitative RT-PCR evaluations were carried out using HE4 (*WFDC2*) and EGFR transcripts from H1975 cells with stable *HE4 shRNA2* expression. *p<0.05; ***p<0.001.

Figure S6 Cell proliferation was measured by an MTT assay in PC-9, H1975, and A549 after combination treatment with *HE4 shRNA2* and gefitinib (**A** and **B**) /osimertinib (**C** and **D**) together or alone for 6 days. ns: p>0.05; *p<0.05; **p<0.01; ***p<0.001; ****p<0.0001.

## Data Availability

The original contributions presented in the study are included in the article/Supplementary Materials.

## Supplementary Materials

The supplementary material is available online at https://doi.org/10.32604/or.2024.04525.
