# Supplementary figures and images for: Knockdown of HE4 suppresses tumor growth and invasiveness in lung adenocarcinoma through regulation of EGFR signaling
Source: Oncol Res. 2024 May 23;32(6):1119–28. doi: 10.32604/or.2024.045025 (PMC11136691; doi:10.32604/or.2024.045025)

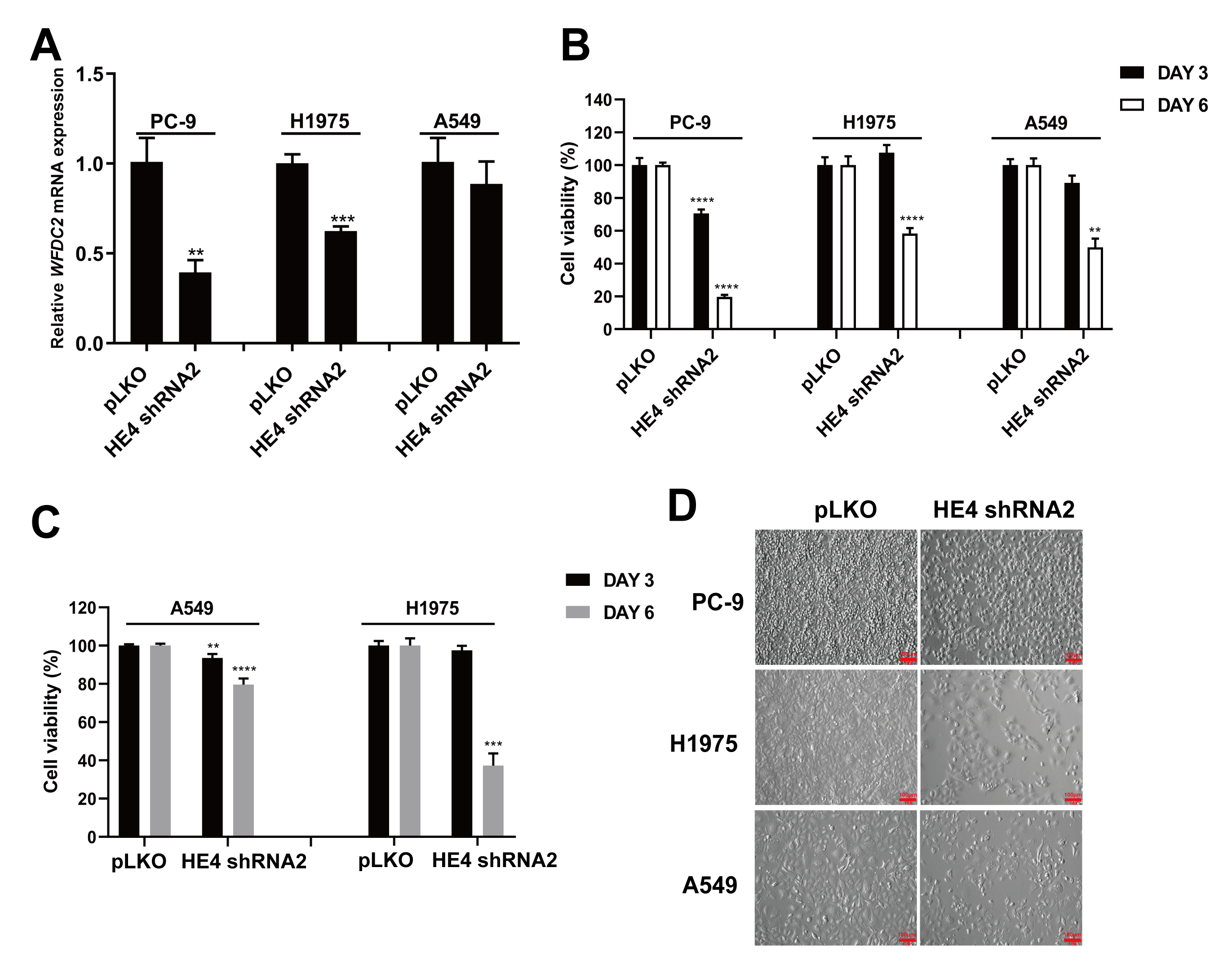

Supplement: Figure S1 [file OncolRes-32-45025-s001.tif]

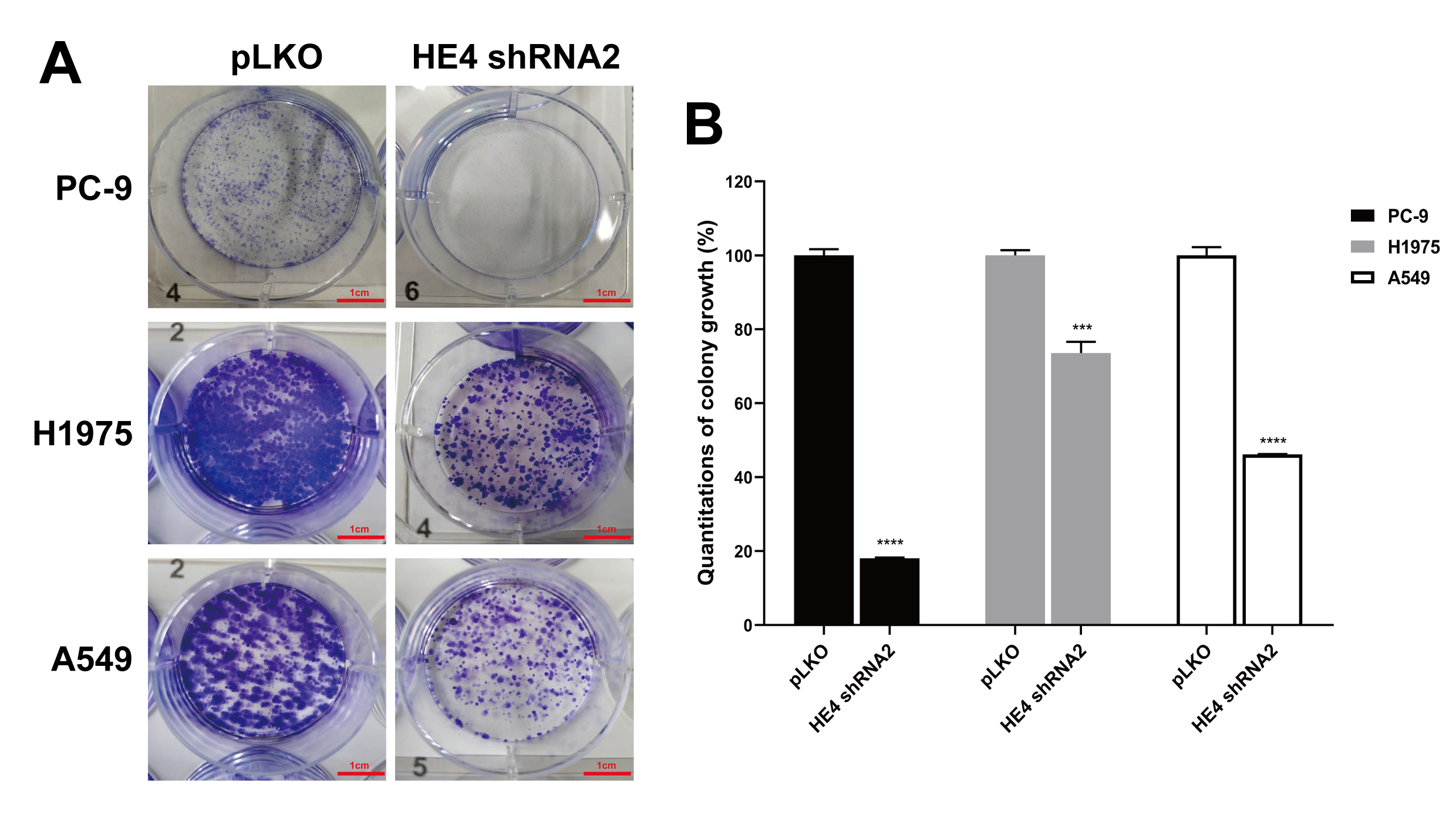

Supplement: Figure S2 [file OncolRes-32-45025-s002.tif]

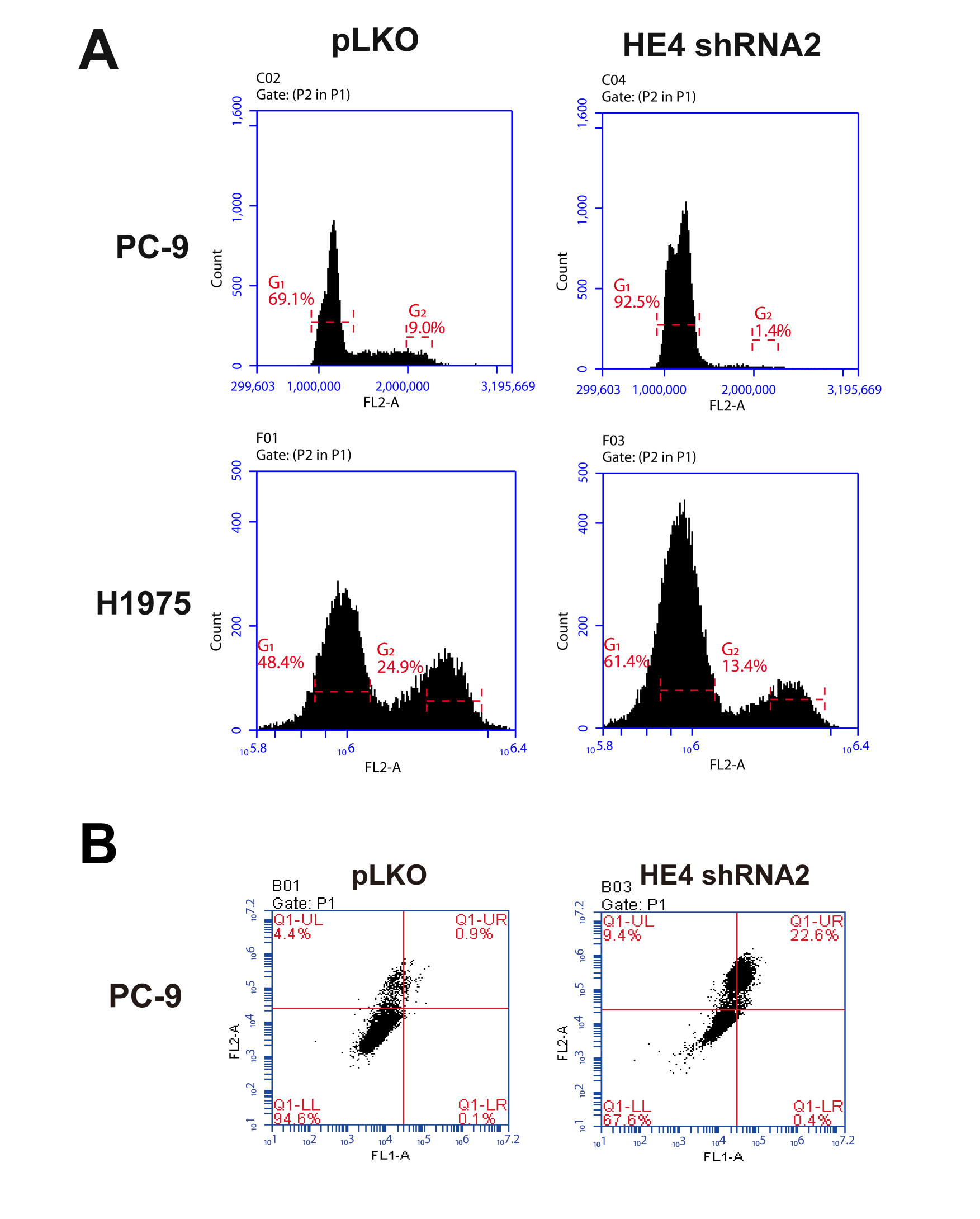

Supplement: Figure S3 [file OncolRes-32-45025-s003.tif]

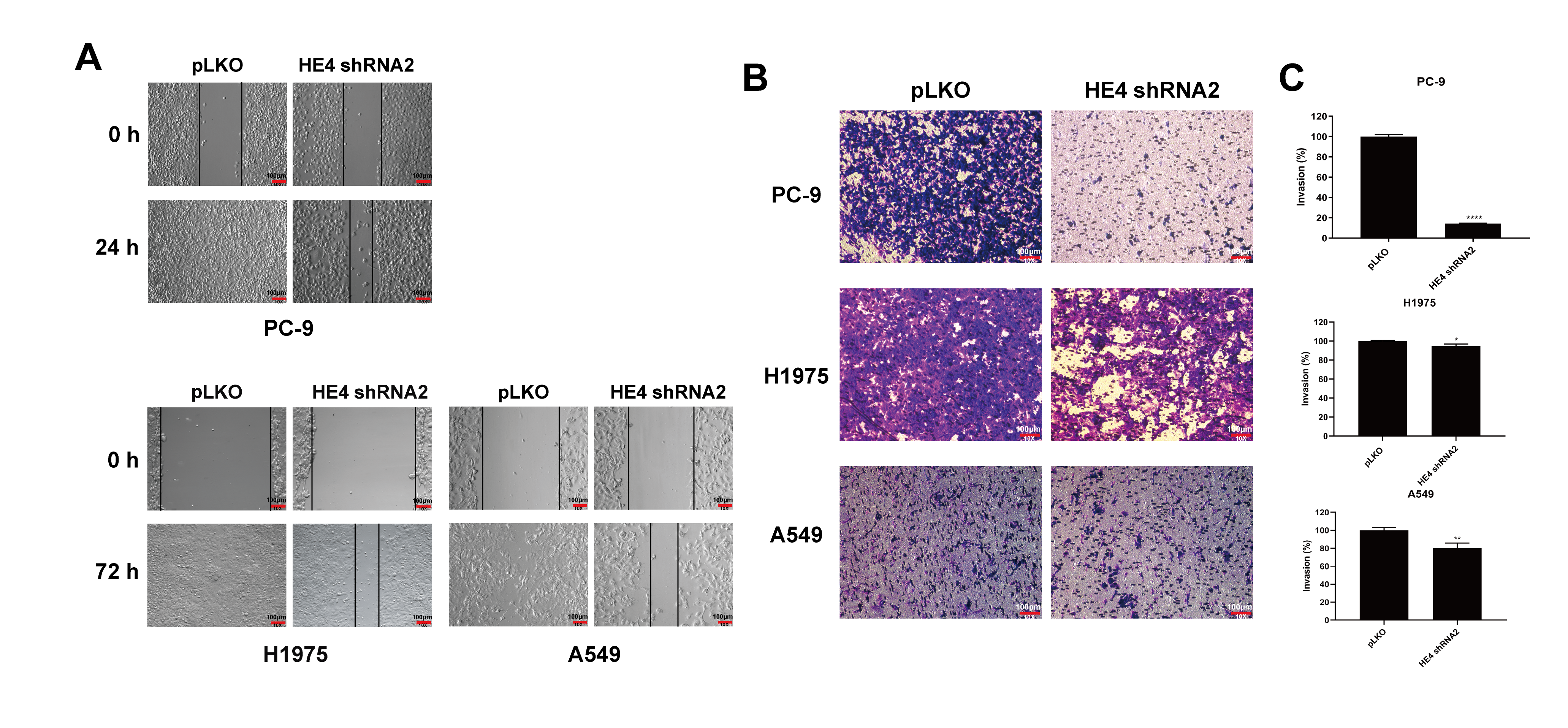

Supplement: Figure S4 [file OncolRes-32-45025-s004.tif]

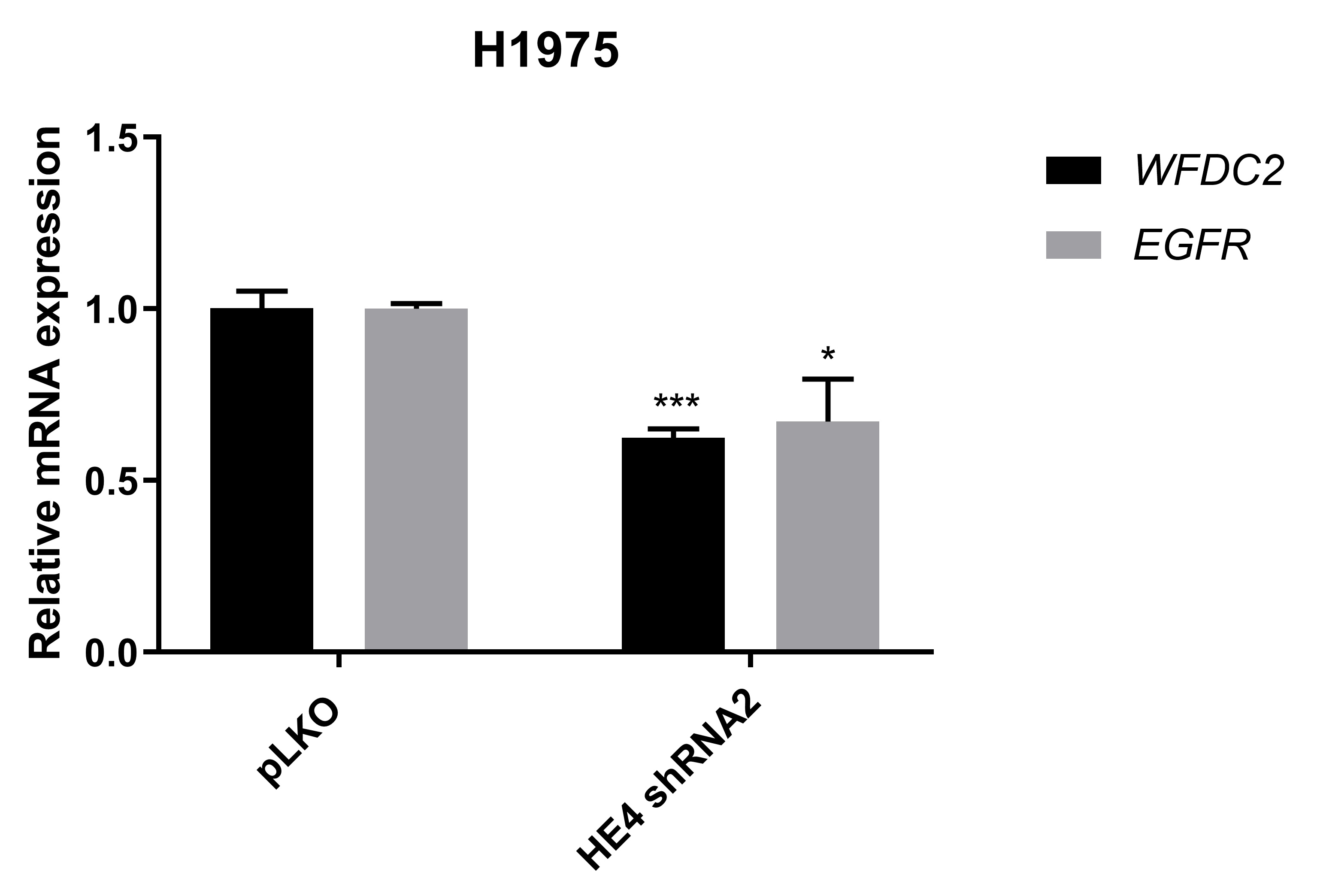

Supplement: Figure S5 [file OncolRes-32-45025-s005.tif]

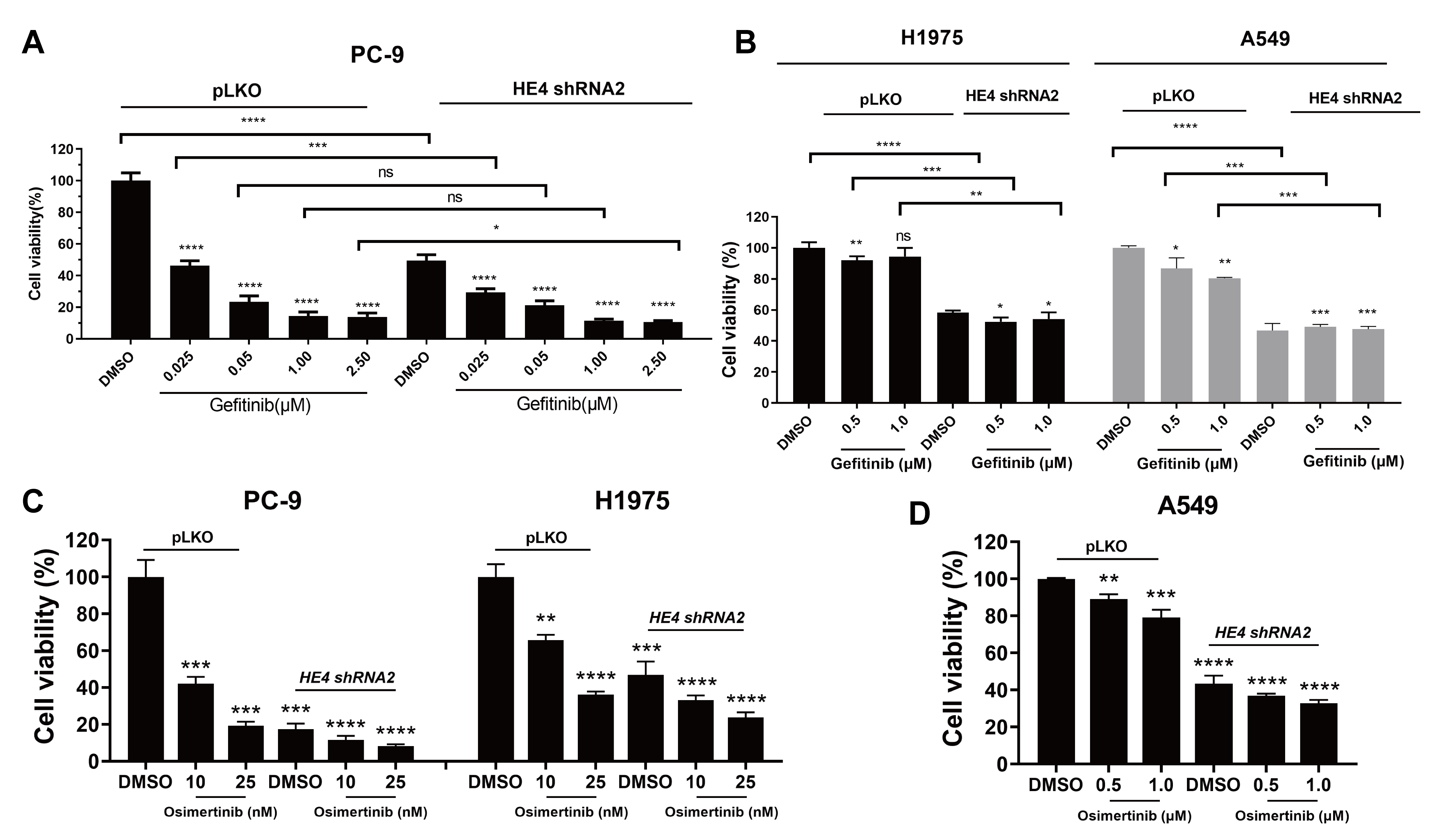

Supplement: Figure S6 [file OncolRes-32-45025-s006.tif]
