# Supplementary material for: Knockdown of HE4 suppresses tumor growth and invasiveness in lung adenocarcinoma through regulation of EGFR signaling
Source: Oncol Res. 2024 May 23;32(6):1119–28. doi: 10.32604/or.2024.045025 (PMC11136691; doi:10.32604/or.2024.045025)
Supplement: Supplementary file 7 [file OncolRes-32-45025-s001.docx]

**Supplementary Table 1 Primary antibody information**

| **Antibodies** | **Catalog number** | **Source** | **Dilution** | **Producer** |
| --- | --- | --- | --- | --- |
| EGFR (western) | sc-03 | Rabbit | 1:500 | Santa Cruz Biotechnology |
| p-EGFR | 2234S | Rabbit | 1:1000 | Cell Signaling Technology |
| p-AKT | 9271S | Rabbit | 1:1000 | Cell Signaling Technology |
| p-MAPK | 9101S | Rabbit | 1:1000 | Cell Signaling Technology |
| GAPDH | abs137959 | Mouse | 1:5000 | Absin Bioscience |
| Actin | abs132001 | Mouse | 1:5000 | Absin Bioscience |
| HE4 (western) |  | Rabbit | 1:500 | Homemade |
| EGFR (co-IP) | sc-120 | Mouse |  | Santa Cruz Biotechnology |
| HE4 (co-IP) | sc-293473 | Mouse |  | Santa Cruz Biotechnology |
